# UX Framework Including Imbalanced UX Dataset Reduction Method for Analyzing Interaction Trends of Agent Systems

**DOI:** 10.3390/s23031651

**Published:** 2023-02-02

**Authors:** Bonwoo Gu, Yunsick Sung

**Affiliations:** 1 Department of Multimedia Engineering, Graduate School, Dongguk University-Seoul, Seoul 04620, Republic of Korea; 2 Department of Multimedia Engineering, Dongguk University-Seoul, Seoul 04620, Republic of Korea

**Keywords:** user experience and user interface, imbalanced UX dataset, artificial intelligence, game agent system, human–computer interaction

## Abstract

The performance of game AI can significantly impact the purchase decisions of users. User experience (UX) technology can evaluate user satisfaction with game AI by analyzing user interaction input through a user interface (UI). Although traditional UX-based game agent systems use a UX evaluation to identify the common interaction trends of multiple users, there is a limit to evaluating UX data, i.e., creating a UX evaluation and identifying the interaction trend for each individual user. The loss of UX data features for each user should be minimized and reflected to provide a personalized game agent system for each user. This paper proposes a UX framework for game agent systems in which a UX data reduction method is applied to improve the interaction for each user. The proposed UX framework maintains non-trend data features in the UX dataset where overfitting occurs to provide a personalized game agent system for each user, achieved by minimizing the loss of UX data features for each user. The proposed UX framework is applied to a game called “Freestyle” to verify its performance. By using the proposed UX framework, the imbalanced UX dataset of the Freestyle game minimizes overfitting and becomes a UX dataset that reflects the interaction trend of each user. The UX dataset generated from the proposed UX framework is used to provide customized game agents of each user to enhanced interaction. Furthermore, the proposed UX framework is expected to contribute to the research on UX-based personalized services.

## 1. Introduction

User experience (UX) [1] technology provides personalized services through user interaction analysis in various fields, such as education and games [2]. UX can be learned through machine learning (ML) to provide personalized services for each user. Furthermore, the deep learning classification algorithm can produce better results than traditional ML in various classification problems [3]. A convolutional neural network (CNN) [4] is a representative non-parametric ML algorithm that shows enhanced classification results by automatically extracting features [5]. However, collecting a large volume of training data in a real environment is cost-prohibitive and time-consuming [6]. In addition, it is difficult to collect data for each user provided quantity limitations as the UX data collected differs from user to user [7]. The limited UX dataset can become an imbalanced dataset [8,9], with overfitting [10] being a frequent occurrence while training non-parametric ML algorithms [11].

In general, in the deep learning field, the overfitting problem associated with an imbalanced dataset is solved with data augmentation [12], considering cost and time issues. Data augmentation is a method to artificially increase data by applying affine transformations [13], such as shift, rotation, and scale, to each class of data [14]. Newly generated data can highlight non-trend data features, thereby playing an important role in minimizing overfitting. However, UX data are generated based on user experience, and general artificial transformation cannot accurately represent user interaction.

For example, in the game field, a vast amount of UX data is collected for analysis and processing to provide an improved agent. However, when analyzing UX data, it is difficult to understand the interaction trend, as the interaction with the game varies for each user. In the traditional game field, there has been a case of inferring and evaluating the user interaction trend using an expert system [15]. In this process, non-trend UX data features are excluded. Thus, to improve the fun of the game by personalizing its difficulty according to user interaction, a method is required to preserve non-trend UX data features and learn an imbalanced dataset without overfitting in a non-parametric ML algorithm.

This paper proposes a UX framework in which a UX data reduction method is applied to improve the interaction of each user in the game agent system. The proposed UX framework introduces data preprocessing, which reduces the UX data, instead of data augmentation, which has a limited application to UX data in learning non-parametric ML algorithms. For the collective reduction of data, the pieces of data with similar features are grouped with an approximate nearest neighbor (ANN) [16] within the same class to select the representative data, thus enabling a reduction in the amount of data in a class with a relatively large amount of data. Therefore, a dataset can be created that preserves the class label without overfitting.

The contributions of this paper are as follows. First, the existing data reduction method has limitations in its application, as it requires users to create an application-dependent data reduction rule. However, the UX data reduction method proposed in this paper is based on the similarity of data, which can be used in other applications with minimal user intervention. Finally, as data reduction is performed while maintaining the UX data features, in what can be an imbalanced UX dataset, it can be used in situations with limitations in the application of data augmentation.

This paper is structured as follows. Section 2 describes related work. Section 3 describes the UX framework for game agent systems using the proposed data reduction method. Section 4 describes the experimental results of applying the proposed UX framework to a game called “Freestyle”. Section 5 draws conclusions from this paper.

## 2. Related Works

This chapter describes the parametric and non-parametric ML algorithms applied in the UX field. In addition, data augmentation and data reduction studies for using an imbalanced dataset without overfitting are described outside of the UX field.

### 2.1. Parametric ML Algorithm in UX

A parametric ML algorithm creates a mathematical model of data trends based on distributional assumptions [17]. As the model is created by estimating the distributional parameters based on the data, a high-performance classification model can be created with a clear data trend despite an imbalanced dataset or a small amount of data. Representative algorithms include the Bayesian estimation (BE) [18], maximum-likelihood estimation (MLE) [19], Markov decision process (MDP) [20], and linear support vector machine (SVM) [21]. Feng et al. built a hierarchical BE model based on the Markov chain Monte Carlo method to evaluate UX and form its related parameters [22]. Biao Wang et al. developed dynamic rumor influence minimization with UX (DRIMUX) based on MLE to minimize malicious rumors and verified the performance by experimenting on large-scale networks [23]. Mengxi Zhang et al. developed a Semi-Markov decision process (SMDP) to manage the power of a mobile device, suggesting a solution that balanced UX and power consumption [24]. Emad Elwany et al. analyzed the user voice UX data and created a model with linear SVM to develop improved voice-enabled personal assistants [25]. Vladimir Nikulin et al. suggested a Markov model that maintained the best of past UX to describe how users interacted with websites [26].

In the parametric ML algorithm, the UX learns parameters suitable for each user from past experience and uses them to provide personalized services. If the user action exhibits an artificial change in its pattern, it can be a means to verify the identity of the user. However, if the past action pattern changes following an improvement in user interaction, the parameters tailored to the user must be recalculated. The parametric ML algorithm has limitations in applying detailed UX changes when changes in difficulty are required due to an improvement in user interaction, like in games.

### 2.2. Non-Parametric ML Algorithm in UX

A non-parametric ML algorithm is a method of modeling data trends similar to the rank method without distributional assumptions [17,27]. Considering its high data dependence, the rank method cannot determine the correct ranking with an imbalanced dataset or an insufficient amount of data. Therefore, the pieces of data simply pushed out of the ranking are ignored and disappear. Representative algorithms include K-nearest neighbors (KNN) [28], neural network [29], Parzen windows [30], and non-linear SVM [31]. Chien-Ming Huang et al. collected UX from humans for physical collaboration with a robot, including object handover, and inferred the current user state through KNN learning [32]. Hong Zeng et al. suggested an AR piano system for improving short-term piano learning for beginners by using an improved UX-based KNN model [33]. Jin Yu-hong et al. suggested a generalized modeling method using the Parzen window technique in an environment with difficulties in acquiring user voices due to limited UX [34]. A. Amanatiadis et al. made a model by learning UX for website properties on a neural network to approximate the relationship between user satisfaction and determinants [35]. Victoria Meza-Kubo et al. evaluated the UX for elderly people using a neural network trained to recognize pleasant and unpleasant emotions with EEG signals. Carmen Bisogni et al. suggested a way to experience a new environment for users by fine-tuning the application to fit the UX of each user using deep neural network (DNN) [36,37]. The non-linear SVM algorithm is an SVM algorithm that can learn with a non-linear kernel using a kernel trick [38] and can solve problems that cannot be solved with a linear kernel. The non-linear kernel includes RBF, POLY, and SIGMOID. Yang Lei et al. suggested a hybrid particle swarm optimization (PSO)-SVM model using network UX to detect abnormal behaviors in network traffic [39].

The non-parametric ML algorithm learns multiple user trends from past UX and is used to improve user interactions through these learned trends or approximate the UX data trend, which is difficult to model mathematically. It is mainly used in fields where sufficient data can be sufficiently collected because an insufficient amount of data leads to incorrect classification results. With a clear data trend, even a small amount of data gives correct results with the rank method but using UX data without a clear trend may lead to overfitting or an incorrect classification. The non-parametric ML algorithm is essential for improving user interaction, such as in situations when setting difficulty as a personalized service in a game, but the real problem in UX data collection is that it often leads to imbalanced datasets. Therefore, there is a need for a method that can learn non-parametric ML algorithms while reflecting the features of non-trend UX data that have been pushed out of the ranking by the rank method.

### 2.3. Data Augmentation and Data Reduction

An imbalance in the training dataset is one of the main problems of ML-based classification. Ling Chen et al. built a neural network that improved the Multi-Label Classification (MLC) performance of DNN based on the relationship between data imbalance and label correlation to enhance the accuracy of labels in non-trend classes [40]. Tianyu Liu et al. proposed hybrid ML to predict stroke based on physiological data with incompleteness and data imbalance, reporting 51.5% less error than other ML-based techniques [41]. In addition to a general affine transformation, various methods have been suggested for data augmentation. Rogez et al. suggested a method to artificially augment a real image dataset with 2D human pose annotations using 3D motion capture data [42]. Terry T. Um et al. improved CNN results by applying rotation, permutation, and time warping to 1D signal data [43]. WEI et al. suggested easy data augmentation (EDA) as a technique for text data [44]. EDA supports synonym replacement, random insertion, random swap, and random deletion to preserve the meaning of sentences and increase the amount of data. Roberta et al. proposed automated data augmentation that could be used in reinforcement learning (RL) [45], reporting an improvement in RL performance by 40% [46]. In the field of natural language processing (NLP) [47], where general affine transformation cannot be applied due to complicated rules, studies on data augmentation are scarce [48] and require additional research. In some applications, a data reduction method suitable for the situation is developed for fast and accurate learning. Jian Zheng et al. used partial mutual information (PMI) [49] and correlation matching-based active learning (CMAL) [50] to suggest a technique to reduce the amount of training data [51]. Gu et al. suggested a method to reduce the amount of training data by grouping the training data of the Gomoku game in similar states together with an ANN [52,53]. This method significantly reduced the amount of training data and induced the DNN to select the next best solution through the action filter.

Data augmentation requires users to develop new rules in addition to general affine transformation depending on the application; furthermore, data reduction methods vary according to the application. When applying traditional data augmentation and traditional data reduction to a UX dataset generally, the user interaction trend may become unclear in the newly created or reduced UX dataset.

## 3. UX Framework of Game Agent System

The performance of game AI plays an important role in the purchase decisions of users [54]. The UX can evaluate the user satisfaction with the AI by analyzing the interaction between the user and the game AI. However, there are many difficulties in the evaluation process of each user-generated UX data, including analyzing the interaction trends of each user [55]. This paper proposes a UX framework that can improve the interaction of each user in game agent systems.

### 3.1. Overview

**Figure 1:**
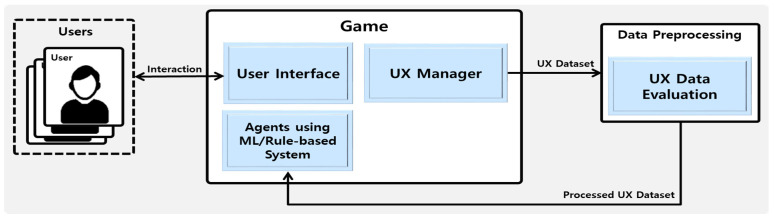
Traditional game agent system based on UX Framework.

The UX-based traditional game agent system framework generally preprocesses the UX dataset using UX evaluation-based approaches [56,57,58], as shown in Figure 1, and applies it to the game agent. The detailed process is as follows. A UX dataset is created by collecting UX data from several users in play interaction through the user interface (UI) in the game. The causes of each piece of UX data are analyzed through UX data evaluation, for example, expert-system-based UX evaluation [56] or a gameplay experience questionnaire (GEQ)-based UX evaluation [57,58]. Based on the analyzed UX data, the game agent system is developed through ML or rule-based systems. Users can be provided with a UX that matches the user interaction trend through the interaction with the developed game agent system.

As UX data are analyzed based on the UX data evaluation results, non-trend UX features are excluded in this process as the trend UX features are emphasized. Therefore, to provide an agent system that reflects the interaction of each user, the UX features for each user must be reflected without distinguishing between trend and non-trend data. As the non-trend dataset contains less data than the trend dataset, the UX dataset can be imbalanced, and overfitting may occur when learning with a non-parametric ML algorithm. In general, to avoid overfitting, non-trend data features are reflected through data augmentation. However, this paper proposes a method to reflect non-trend UX features using a UX data reduction method as artificial UX data transformation does not match the user interaction trend. The proposed UX data reduction method can avoid overfitting.

**Figure 2:**
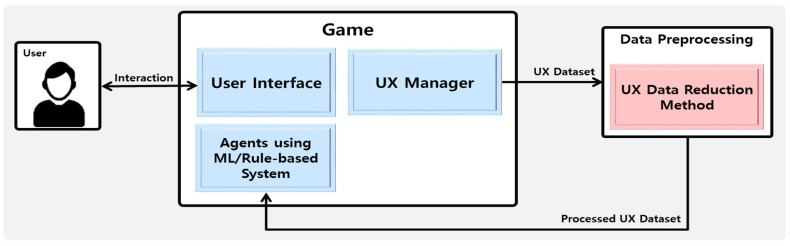
Proposed UX framework including UX data reduction method for game agent AI system.

The UX framework for game agent systems proposed by this paper is shown in Figure 2. The proposed UX-based game agent system improves the UX by providing a personalized agent system for each user. This system is configured using only individual UX data to highlight the UX features of individual users. In the UX data reduction method proposed by this paper, a processed UX dataset reflecting trend UX features and non-trend UX features for each user is generated through the proposed ANN-based clustering method and used for the game agent.

### 3.2. UX Data Reduction Method

**Figure 3:**
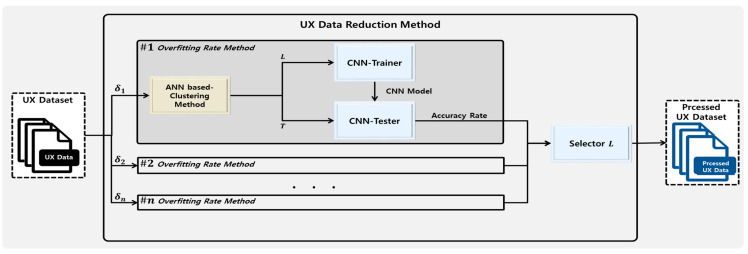
UX data reduction method framework.

Figure 3 shows the framework of the UX data reduction method, which determines the ANN distance of the ANN-based clustering method with  δ, meaning the maximum similarity. As the results of the ANN-based clustering method depend on δ, various heuristic values are applied to n ANN-based clustering methods, and the results of the ANN-based clustering method with the highest accuracy rate are used. Trainset L and Testset T are created using the UX dataset through the ANN-based clustering method, given that Trainset L is used for learning and Testset T for evaluating accuracy by CNN.

The received UX dataset is processed as follows to derive a UX dataset with a low probability of overfitting. When the CNN model trained with Trainset L is evaluated for accuracy with Testset T, a low accuracy means that Trainset L has a high probability of overfitting. In contrast, a high accuracy means that Trainset L has a low probability of overfitting. Following the accuracy assessment of the CNN results obtained by inputting the heuristic δ, Trainset L of δ, with the highest accuracy, is selected as the processed UX dataset. 

### 3.3. ANN-Based Clustering Method

**Figure 4:**
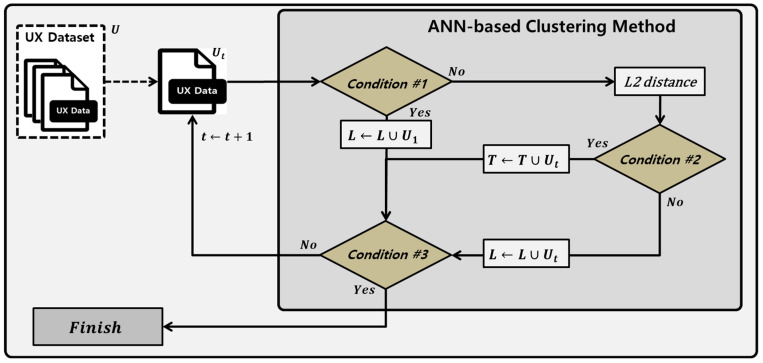
Flowchart of ANN-based clustering method.

As the UX dataset for each user can be an imbalanced dataset, overfitting may occur when learning without preprocessing. In this paper, the amount of data is reduced by grouping similar UX data within the same class and selecting representative data based on the similarity calculated by ANN [52,53]. Figure 4 shows the flowchart of the ANN-based clustering method.

U is the input UX dataset, and $U_{t}$ is the t-th element of the UX dataset U that can learn nonparametric ML algorithms. $L_{i}$ means the i-th element of Trainset L. The ANN-based clustering method uses the L2 distance to execute the ANN algorithm as follows: if Trainset L is ∅ as condition #1, Trainset L is added by UX data $U_{1}$. Otherwise, the similarities of all pairs of $U_{t}$ and $L_{i}$ are calculated using the L2 distance. $L^{*}$ is the $L_{i}$ with minimum distance; if $U_{t}$ and $L^{*}$ in the same class have a similarity smaller than or equal to δ as condition #2, $U_{t}$ is added to Testset T. If $U_{t}$ and $L^{*}$ are in different classes or have a similarity larger than δ, $U_{t}$ is added to Trainset L; ANN-based clustering method is repeated until all U elements in the UX dataset as condition #3 are classified into Trainset L or Testset T.

Trainset L, created through the ANN-based clustering method, preserves the classes for each UX dataset. The data of a class with a large amount of data can be reduced to change an imbalanced dataset to a balanced dataset. Accordingly, the ANN-based clustering method can reflect trend UX features and non-trend UX features to minimize overfitting. 

**Figure 5:**
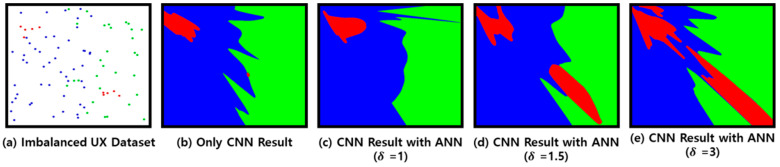
CNN results of imbalanced dataset.

Figure 5 shows an example of the CNN result obtained by learning Trainset L created by the ANN-based clustering method where each cluster has own color. Figure 5a shows an example of a UX dataset, where an imbalance is assumed. Figure 5b shows the result of learning Figure 5a with CNN. Figure 5c–e show the results of training with Trainset L created by the ANN-based clustering method according to δ by CNN.

As Trainset L, created through the ANN-based clustering method, groups similar data within the same class and selects representative data, different results are shown according to δ in general. As shown in Figure 5c, when δ is small, data reduction is not performed, and Trainset L is similar to Figure 5a. As shown in Figure 5e, when δ is large, the features of the UX data to be maintained are also grouped, which creates an error in the CNN result. As shown in Figure 5d, when δ is applied, the UX dataset is created without overfitting, showing high classification results. Thus, the UX data reduction method uses CNN to determine the optimal δ according to the accuracy.

## 4. Experiments

The UX framework for the game agent system proposed in this paper is applied to a Freestyle game, and whether the UX data reduction method reflects the non-trend UX feature in the imbalanced dataset is verified. Section 4.1 describes the Freestyle game and the experimental environment applied. Section 4.2 and Section 4.3 evaluate the performance of the proposed UX framework for the game agent system.

### 4.1. Experimental Environment

The Freestyle game [59] is a PC online game developed using the motif of 3-on-3 street basketball. Because each agent in the game was developed using a rule-based system called a finite state machine (FSM) [60], realizing an agent that provides customized movements when considering the user interactions is limited. Thus, the actions of the agent must be determined and executed while reflecting the UX. However, although catching and moving actions are frequently conducted owing to the nature of the game, shooting and rebounding actions are applied less frequently. Because the actions conducted by each user differ, the UX data becomes an imbalanced dataset, which causes frequent overfittings in non-parametric ML algorithms during the learning process.

**Figure 6:**
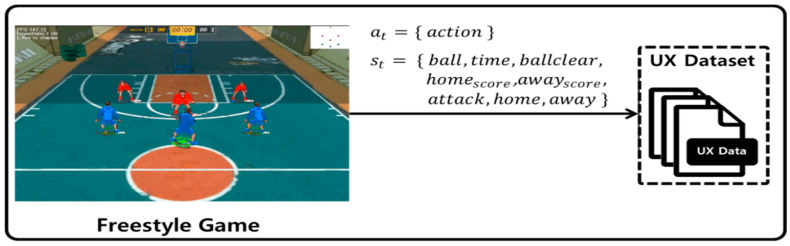
UX dataset of Freestyle game.

As shown in Figure 6, the UX data of the Freestyle game are represented by action $a_{t}$ entered by the user through the UI and by the state $s_{t}$ of the Freestyle game.

Action $a_{t}$, which refers to an action command entered by the user to the agent from among shooting, passing, fast breaking, catching, moving, rebounding, call-passing, stealing, and blocking, is input. State $s_{t}\;$ is composed as follows. Here, ball refers to the 3D location of the basketball, and time refers to the duration of the game. In the Freestyle game, an event called a ballclear occurs. In a ballclear state, the team in possession of the ball must move the ball to the 3-point shooting area. Because shooting is impossible, it is favorable to attack and move the ball to the 3-point shooting area as quickly as possible. Some users move the ball to the 3-point shooting area as quickly as they can by passing or dribbling for a quick ballclear. Here, ballclear indicates which team, home or away, is in a ballclear state. In addition, $home_{score}\;$ indicates the score of the home team, and $away_{score}\;$ indicates the score of the away team. Moreover, attack indicates which team is in possession of the ball, and home and away contain the main state and 3D location information of home and away team agents, respectively. Main state indicates whether the agent is shooting or passing. 

In this paper, the Freestyle game dataset was collected from 10 random users in online environments. Instead of a UX data evaluation that directly requires manual work, the frequency of action occurrence is analyzed in UX data, and unintended actions by users in the imbalanced UX dataset of individuals are also treated and analyzed in terms of frequency of occurrence. If the user’s intent is not clearly included in the UX data, it acts as noise to analyze the user’s trend in the proposed framework. In these experiments, out of 10 users, the experiment was conducted based on 3 users whose frequency of action was clearly identified in the UX data. User #1 mainly acts under a goal post and catches a ball when the ball of the Freestyle game enters a *Loose Ball* state. User #2 primarily acts around the 3-point line and performs long shots. User #3 mainly blocks the number of attacks by opponent agents.

The input layer has 37 neurons according to the state $s_{t}$. Given that the action $a_{t}$ is converted into a one-hot-encoding based vector, the output layer has nine neurons. Considering states and actions, the CNN used in this paper has two convolution layers with 5 × 5 kernel and 3 × 3 kernel, and two max pooling layers. There are four hidden layers with 128, 512, 256, and 64 neurons, respectively. 

The experiment described in this experiment was developed using C/C++, Python 3.8, and TensorFlow 2.0. CPU I-7, 16 GB of RAM, and an NVIDA GeForce GTX 1650 were used in the experimental environment.

### 4.2. UX Dataset Analysis of Freestyle Game

**Figure 7:**
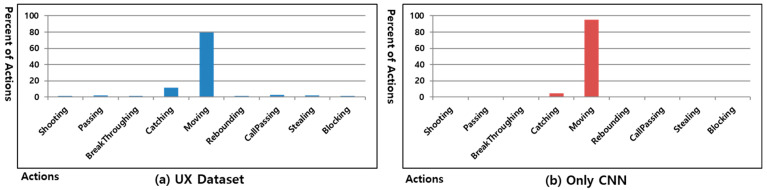
Percent of actions in UX dataset of user #1 and using only CNN in the Freestyle game.

To evaluate the performance of the proposed UX Framework, we analyzed the UX dataset of the Freestyle game and analyzed whether the interaction trend for each user is maintained when learning the UX Dataset from the non-parametric ML algorithm. Figure 7 shows the action ratio in the UX Dataset of user #1 generated by playing the Freestyle game and the action ratio when processed using a CNN.

Figure 7a shows the action ratio of the UX dataset of user #1 generated when the Freestyle game was played 30 times. The UX dataset of user #1 shown in Figure 7a becomes an imbalanced UX dataset when reflecting the characteristics of the Freestyle game and user interactions. Figure 7b shows the action ratio of 30 games after learning the UX dataset of user #1 of Figure 7a using only a CNN. In the UX dataset of user #1 shown in Figure 7a, an overfitting occurred because the percentage of actions other than catching and moving was less than 8.8%. CNN is designed to be weak in imbalanced data sets. There is a limit to solving imbalanced data sets by multiple convolution layers and pooling layers [61]. As shown in Figure 7b, no actions except catching and moving were conducted. In the UX dataset of user #1 shown in Figure 7a, the minimum action rate has 0.16% blocking. If it becomes a balanced UX dataset having 0.16% of the total UX dataset for each action in training set L created using an ANN-based clustering method according to the **δ** value, the generated training set L will have a ratio of 1.44% of the total UX dataset. However, because the distribution of UX data in the UX dataset is also different for each user owing to varying user interactions, it is difficult to ensure that the balanced UX dataset, which has 0.16% of the total UX dataset for each action, reflects the user interaction trend without the occurrence of an overfitting.

### 4.3. Results of Proposed UX Framework

In this section, the performance of the processed UX dataset created through the UX framework for the game agent system is evaluated. In this paper, to check the performance of training set L generated using the ANN-based clustering method according to the δ value, training set L with the highest accuracy rate applied to a CNN was selected as the processed UX dataset. Table 1 shows the ratio of training set L and test set T generated through the ANN-based clustering method according to the value of **δ** and the accuracy rate of the CNN of user #1.

As shown in Table 1 (a) and Table 1 (b), if δ is small, the training set L will take up a small proportion compared to the entire UX dataset; however, because it can still become an imbalanced UX dataset, an error occurs in the CNN result. As shown in Table 1 (d) through Table 1 (f), if δ is large, only one UX data for every action in training set L is contained, and the UX data to be maintained are also reduced, resulting in an error in the CNN result. When δ is applied as shown in Table 1 (c), the highest CNN accuracy rate is obtained, and the amount of training data is also reduced to 0.47% of the total UX dataset. It is also expected that the δ value minimizes an overfitting and maintains the interaction trend for each user.

**Figure 8:**
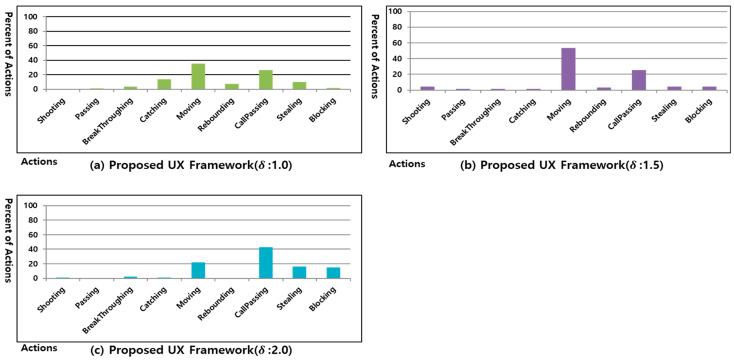
Percentage of actions of proposed UX framework in Freestyle game of user #1.

Figure 8 shows the results of learning the processed UX dataset of user #1 of the UX data reduction method, according to the heuristic δ in the CNN. 

Figure 8a–c show the percentage of actions of 30 games after training the processed UX dataset based on the UX data reduction method according to the δ value when using a CNN based on UX dataset of user #1. As shown in Figure 8a, when δ is small, some of the trends in Figure 7a are maintained when the percentage of an action has the highest value, such as moving. However, the other actions behave in a different way from that shown in Figure 7a. As shown in Figure 8c, when δ is large, reducing more UX data than necessary results in a different trend from that of Figure 7a. When δ is applied, as shown in Figure 8b, the action percentage trend in Figure 8b becomes similar to that of Figure 7a, except for actions such as catching and call passing. In addition, although shooting in Figure 8a and rebounding in Figure 8c are never conducted, it can be confirmed that all actions are applied in Figure 8b. Through this experiment, when δ is 1.5 in the Freestyle game, the interaction by the user can be reflected while minimizing the overfitting. 

**Figure 9:**
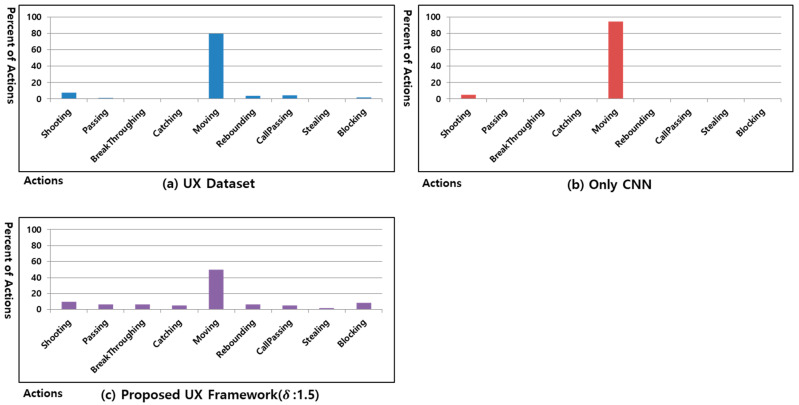
Percentage of actions of proposed UX framework in Freestyle game of user #2.

Figure 9 shows the results of learning the processed UX dataset in the CNN based on the UX dataset of user #2. Figure 9a shows the action ratio of the UX dataset generated when the UX dataset of user #2 of the Freestyle game was played 30 times. Figure 9b shows the action ratio of 30 games after learning the UX dataset of Figure 9a using only a CNN. Figure 9c is the result of learning the processed UX dataset with δ set to 1.5 based on UX dataset of user #2. Figure 9b does not execute actions except for shooting and moving. However, Figure 9c maintains the trend of Figure 9a and performs other actions. 

**Figure 10:**
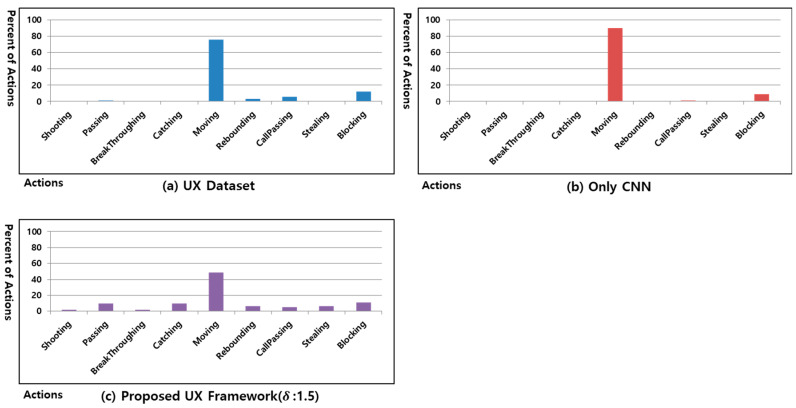
Percentage of actions of proposed UX framework in Freestyle game of user #3.

Figure 10 shows the results of learning the processed UX dataset in the CNN based on the UX dataset of user #3. Figure 10a shows the action ratio of the UX Dataset generated when the UX dataset of user #3 of the Freestyle game was played 30 times. Figure 10b shows the action ratio of 30 games after learning the UX Dataset of Figure 10a using only a CNN. Figure 10c is the result of learning the processed UX dataset with δ set to 1.5, based on the UX dataset of user #3. Figure 10b does not perform actions except for moving, blocking, and call passing. However Figure 10c maintains the trend of Figure 10a and performs other actions. 

The proposed UX framework described in this paper minimizes an overfitting and generates a processed UX dataset to reflect the user interaction trend. The generated processed UX dataset can be used for the customized game agents of each user, thereby improving user interaction trends. 

There is the possibility to produce some actions that are different from the action based on the user’s trend. For example, the percentages of the non-trend actions in Figure 8b, Figure 9c, and Figure 10c by the proposed method were different from the percentages of the non-trend actions in Figure 7a, Figure 9a, and Figure 10a by the UX dataset of each user.

## 5. Conclusions

In this paper, the representative UX data for each class are selected by the ANN-based clustering method for the imbalanced UX dataset for each user, where overfitting occurs. This process changes an imbalanced UX dataset to a balanced UX dataset to minimize overfitting and provides a game agent system that reflects the interaction of each user for each user. As the results of the ANN-based clustering method depend on δ, a heuristic δ suitable for CNN was selected. The proposed UX framework has been tested by applying it to the Freestyle game.

The UX dataset created in the Freestyle game becomes an imbalanced UX dataset by the feature of the game and the interaction trend by the user, and overfitting occurred in CNN learning. In order to select a reasonable one, the Trainset Testset generated by the ANN-based-clustering method was applied to CNN to select the value of the highest accuracy rate. Experiments showed that the accuracy rate was the highest when the value was 1.5, and that the action ratio was the most similar to that of the UX dataset generated in the Freestyle game. 

The proposed UX framework minimizes the loss of UX data features for each user. In the future, a further paper will be conducted on how to evaluate the UX by creating a UX evaluation model for each user with the processed UX dataset created through the UX data reduction method. The proposed UX framework is expected to contribute to the research into UX-based personalized services.

## Figures and Tables

**Table 1:** Results of CNN—of ANN-based clustering methods of user #1.

	Trainset L	Testset T	Accuracy Rate
(a) Proposed UX Framework (δ:0.5)	8.62% of UX Dataset	91.38% of UX Dataset	62.5%
(b) Proposed UX Framework (δ:1.0)	6.42% of UX Dataset	93.58% of UX Dataset	65.5%
(c) Proposed UX Framework (δ:1.5)	0.47% of UX Dataset	99.53% of UX Dataset	78.3%
(d) Proposed UX Framework (δ:2.0)	0.023% of UX Dataset	99.97% of UX Dataset	66.9%
(e) Proposed UX Framework (δ:2.5)	0.023% of UX Dataset	99.97% of UX Dataset	66.9%
(f) Proposed UX Framework (δ:3.0)	0.023% of UX Dataset	99.97% of UX Dataset	66.9%

## Data Availability

Not applicable.

## References

[1] Bernhaupt R. (2015). Game User Experience Evaluation.

[2] Maier M., Marouane C., Elsner D. DeepFlow: Detecting Optimal User Experience from Physiological Data Using Deep Neural Networks. Proceedings of the 18th International Conference on Autonomous Agents and MultiAgent Systems.

[3] Wang P., Fan E., Wang P. (2020). Comparative analysis of image classification algorithms based on traditional machine learning and deep learning. Pattern Recognit. Lett..

[4] O’shea K., Nash R. (2015). An introduction to convolutional neural networks. arXiv.

[5] Li P., Chen Z., Yang L.T., Zhang Q., Deen M.J. (2017). Deep Convolutional Computation Model for Feature Learning on Big Data in Internet of Things. IEEE Trans. Ind. Inform..

[6] Shorten C., Khoshgoftaar T.M. (2019). A survey on Image Data Augmentation for Deep Learning. J. Big Data.

[7] Santoso H.B., Schrepp M. (2019). The impact of culture and product on the subjective importance of user experience aspects. Heliyon.

[8] Lemaître G., Nogueira F., Aridas C.K. (2017). Imbalanced-learn: A python toolbox to tackle the curse of imbalanced datasets in machine learning. J. Mach. Learn. Res..

[9] Wang S., Liu W., Wu J., Cao L., Meng Q., Kennedy P.J. Training deep neural networks on imbalanced data sets. Proceedings of the 2016 International Joint Conference on Neural Networks (IJCNN).

[10] Cogswell M., Ahmed F., Girshick R., Zitnick L., Batra D. (2015). Reducing overfitting in deep networks by decorrelating representations. arXiv.

[11] Li Z., Kamnitsas K., Glocker B. (2020). Analyzing Overfitting Under Class Imbalance in Neural Networks for Image Segmentation. IEEE Trans. Med Imaging.

[12] Fawzi A., Samulowitz H., Turaga D., Frossard P. Adaptive data augmentation for image classification. Proceedings of the 2016 IEEE International Conference on Image Processing (ICIP).

[13] Cireşan D.C., Meier U., Masci J., Gambardella L.M., Schmidhuber J. (2011). High-performance neural networks for visual object classification. arXiv.

[14] Perez L., Wang J. (2017). The effectiveness of data augmentation in image classification using deep learning. arXiv.

[15] Gu W., Foster K., Shang J., Wei L. (2019). A game-predicting expert system using big data and machine learning. Expert Syst. Appl..

[16] Arya S., Mount D.M. (1993). Approximate nearest neighbor queries in fixed dimensions. SODA.

[17] Altman D.G., Bland J.M. (2009). Parametric v non-parametric methods for data analysis. BMJ.

[18] Kruschke J.K. (2013). Bayesian estimation supersedes the t test. J. Exp. Psychol. Gen..

[19] Myung I.J. (2003). Tutorial on maximum likelihood estimation. J. Math. Psychol..

[20] Levin E., Pieraccini R., Eckert W. Using Markov decision process for learning dialogue strategies. Proceedings of the 1998 IEEE International Conference on Acoustics, Speech and Signal Processing, ICASSP’98 (Cat. No. 98CH36181).

[21] Suthaharan S. (2016). Support vector machine. Machine Learning Models and Algorithms for Big Data Classification.

[22] Zhou F., Lei B., Liu Y., Jiao R.J. (2017). Affective parameter shaping in user experience prospect evaluation based on hierarchical Bayesian estimation. Expert Syst. Appl..

[23] Wang B., Chen G., Fu L., Song L., Wang X. (2017). DRIMUX: Dynamic Rumor Influence Minimization with User Experience in Social Networks. IEEE Trans. Knowl. Data Eng..

[24] Zhang M., Li Y., Chen H. A semi-Markov decision process based dynamic power management for mobile devices. Proceedings of the 2016 IEEE International Conference on Real-time Computing and Robotics (RCAR).

[25] Elwany E., Shakeri S. (2014). Enhancing Cortana user experience using machine learning. Recall.

[26] Nikulin V., Smola A.J. Parametric model-based clustering. Proceedings of the Data Mining, Intrusion Detection, Information Assurance, and Data Networks Security 2005.

[27] Breitling R., Herzyk P. (2005). Rank-based methods as a non-parametric alternative of the T-statistic for the analysis of biological microarray data. J. Bioinform. Comput. Biol..

[28] Kramer O. (2013). K-nearest neighbors. Dimensionality Reduction with Unsupervised Nearest Neighbors.

[29] Wang S.-C. (2003). Artificial neural network. Interdisciplinary Computing in Java Programming.

[30] Jain A.K., Ramaswami M.D. (1988). Classifier design with Parzen windows. Machine Intelligence and Pattern Recognition.

[31] Azimi-Pour M., Eskandari-Naddaf H., Pakzad A. (2019). Linear and non-linear SVM prediction for fresh properties and compressive strength of high volume fly ash self-compacting concrete. Constr. Build. Mater..

[32] Huang C.-M., Cakmak M., Mutlu B. Adaptive Coordination Strategies for Human-Robot Handovers. Proceedings of the Robotics: Science and Systems.

[33] Zeng H., He X., Pan H. (2019). A New Practice Method Based on KNN Model to Improve User Experience for an AR Piano Learning System. International Conference on Human-Computer Interaction.

[34] Jin Y.-H. (2012). Short speaker verification based on Parzen window estimation. J. Chin. Comput. Syst..

[35] Amanatiadis A., Mitsinis N., Maditinos D. (2015). A neural network-based approach for user experience assessment. Behav. Inf. Technol..

[36] Meza-Kubo V., Morán A.L., Carrillo I., Galindo G., García-Canseco E. (2016). Assessing the user experience of older adults using a neural network trained to recognize emotions from brain signals. J. Biomed. Inform..

[37] Bisogni C., Cascone L., Castiglione A., Passero I. (2021). Deep learning for emotion driven user experiences. Pattern Recognit. Lett..

[38] Hofmann M. (2006). Support vector machines-kernels and the kernel trick. Notes.

[39] Lei Y. Network anomaly traffic detection algorithm based on SVM. Proceedings of the 2017 International Conference on Robots & Intelligent System (ICRIS).

[40] Chen L., Wang Y., Li H. (2022). Enhancement of DNN-based multilabel classification by grouping labels based on data imbalance and label correlation. Pattern Recognit..

[41] Liu T., Fan W., Wu C. (2019). A hybrid machine learning approach to cerebral stroke prediction based on imbalanced medical dataset. Artif. Intell. Med..

[42] Rogez G., Schmid C. Mocap-guided data augmentation for 3D pose estimation in the wild. Proceedings of the 29th International Conference on Neural Information Processing Systems.

[43] Um T.T., Pfister F.M.J., Pichler D., Endo S., Lang M., Hirche S., Fietzek U., Kulić D. Data augmentation of wearable sensor data for parkinson’s disease monitoring using convolutional neural networks. Proceedings of the 19th ACM International Conference on Multimodal Interaction.

[44] Wei J., Zou K. (2019). Eda: Easy data augmentation techniques for boosting performance on text classification tasks. arXiv.

[45] Kaelbling L.P., Littman M.L., Moore A.W. (1996). Reinforcement learning: A survey. J. Artif. Intell. Res..

[46] Raileanu R., Goldstein M., Yarats D., Kostrikov I., Fergus R. (2020). Automatic data augmentation for generalization in deep reinforcement learning. arXiv.

[47] Chowdhary K. (2020). Natural language processing. Fundamentals of Artificial Intelligence.

[48] Liu P., Wang X., Xiang C., Meng W. A survey of text data augmentation. Proceedings of the 2020 International Conference on Computer Communication and Network Security (CCNS).

[49] Frenzel S., Pompe B. (2007). Partial Mutual Information for Coupling Analysis of Multivariate Time Series. Phys. Rev. Lett..

[50] Li X., Zheng J. (2016). Active Learning for Regression with Correlation Matching and Labeling Error Suppression. IEEE Signal Process. Lett..

[51] Zheng J., Yang W., Li X. Training data reduction in deep neural networks with partial mutual information based feature selection and correlation matching based active learning. Proceedings of the 2017 IEEE International Conference on Acoustics, Speech and Signal Processing (ICASSP).

[52] Gu B., Sung Y. (2021). Enhanced Reinforcement Learning Method Combining One-Hot Encoding-Based Vectors for CNN-Based Alternative High-Level Decisions. Appl. Sci..

[53] Gu B., Sung Y. (2021). Enhanced DQN Framework for Selecting Actions and Updating Replay Memory Considering Massive Non-Executable Actions. Appl. Sci..

[54] Nareyek A. (2004). AI in Computer Games: Smarter games are making for a better user experience. What does the future hold?. Queue.

[55] Bernhaupt R., Mueller F.F. Game user experience evaluation. Proceedings of the 2016 CHI Conference Extended Abstracts on Human Factors in Computing Systems.

[56] Kaosar R.N., Murtadha I., Shahbodin F., Riza L.S. (2021). Expert system using the educational game to determine children’s autism levels using forward chaining. Linguist. Cult. Rev..

[57] Law E.L.-C., Brühlmann F., Mekler E.D. Systematic review and validation of the game experience questionnaire (geq)-implications for citation and reporting practice. Proceedings of the 2018 Annual Symposium on Computer-Human Interaction in Play.

[58] Engl S., Nacke L.E. (2013). Contextual influences on mobile player experience—A game user experience model. Entertain. Comput..

[59] (2004). Freestyle. Windows PC, Joycity Corp. https://fs.joycity.com/web/main.do.

[60] Rostianingsih S., Satiabudhi G., Wijaya H.K. (2013). Game Simulasi Finite State Machine Untuk Pertanian dan Peternakan. Ph.D. Thesis.

[61] Valova I., Harris C., Mai T., Gueorguieva N. (2020). Optimization of Convolutional Neural Networks for Imbalanced Set Classification. Procedia Comput. Sci..

